# Profiles of physical, emotional and psychosocial wellbeing in the Lothian birth cohort 1936

**DOI:** 10.1186/1471-2318-12-64

**Published:** 2012-10-22

**Authors:** Andrea R Zammit, John M Starr, Wendy Johnson, Ian J Deary

**Affiliations:** 1 Department of Psychology, Centre for Cognitive Aging and Cognitive Epidemiology, University of Edinburgh, Edinburgh, EH8 9J, UK; 2 Geriatric Medicine Unit, Centre for Cognitive Aging and Cognitive Epidemiology, University of Edinburgh, Edinburgh, EH8 9JZ, UK; 3 Department of Psychology, Centre for Cognitive Aging and Cognitive Epidemiology, University of Edinburgh, Edinburgh, EH8 9JZ, UK; 4 Department of Psychology, Centre for Cognitive Aging and Cognitive Epidemiology, University of Edinburgh, 7 George Square, Edinburgh, EH8 9JZ, Scotland, UK

**Keywords:** Physical wellbeing, Psychosocial wellbeing, Profiles, Latent class analysis

## Abstract

**Background:**

Physical, emotional, and psychosocial wellbeing are important domains of function. The aims of this study were to explore the existence of separable groups among 70-year olds with scores representing physical function, perceived quality of life, and emotional wellbeing, and to characterise any resulting groups using demographic, personality, cognition, health and lifestyle variables.

**Methods:**

We used latent class analysis (LCA) to identify possible groups.

**Results:**

Results suggested there were 5 groups. These included High (n = 515, 47.2% of the sample), Average (n = 417, 38.3%), and Poor Wellbeing (n = 37, 3.4%) groups. The two other groups had contrasting patterns of wellbeing: one group scored relatively well on physical function, but low on emotional wellbeing (Good Fitness/ Low Spirits,n = 60, 5.5%), whereas the other group showed low physical function but relatively well emotional wellbeing (Low Fitness/Good Spirits, n = 62, 5.7%). Salient characteristics that distinguished all the groups included smoking and drinking behaviours, personality, and illness.

**Conclusions:**

Despite there being some evidence of these groups, the results also support a largely one-dimensional construct of wellbeing in old age—for the domains assessed here—though with some evidence that some individuals have uneven profiles.

## Background

Good physical functioning and emotional and psychosocial wellbeing help in maintaining overall wellbeing in old age; indeed, good functioning in one of these domains is often associated with good functioning in the others [1-7]. Active engagement and coping with age-related challenges such as functional limitations, physical disability or dependence on others, which are associated with feelings of depression, may reflect good social resources and successful psychosocial adaptation, including strategising and positive attitudes [1-10].

Given the importance of different aspects of wellbeing, interest surrounds the question of whether physical, psychosocial and emotional domains of wellbeing generally relate closely, or whether the domains are independent of each other. The common cause hypothesis, first applied to cognition and sensory functions [11] suggests that functioning in old age lies on a single spectrum ranging from low to high, whereby individuals fall into high, medium, or low functioning groups in general, across domains of wellbeing [12-14]. With this hypothesis, the existence of uneven aging profiles and any associations between these and other important variables, such as personality traits, is not emphasized [15]. Another hypothesis is that only some individuals show good or poor wellbeing consistently across physical and psychosocial aspects of wellbeing, whereas others are good in one and poor in another. Such unevenness across wellbeing domains might reflect a mixture of gains and losses brought about by age and by different causes, and affecting individuals in different ways [16-18]. The wide variability in health, personality and social attitudes that exists throughout the life-span may contribute to uneven and numerous profiles of wellbeing in old age [19,20]. Better cognitive skills and higher positive personality traits, such as high Emotional Stability, high Conscientiousness, high Extraversion, high Openness and high Agreeableness, are associated with higher quality physical, social and emotional wellness [6,19]. Hence, different wellbeing profiles might associate with more stable traits, such as personality and cognitive functioning [21].

The first aim of the present study was to characterise the profiles of physical, emotional and psychosocial wellbeing that could be identified in groups in 70-year-old individuals of the Lothian Birth Cohort 1936 [22], including levels of physical functioning, quality of life, and emotional stability as the grouping variables. The second aim was to identify external variables—measures other than those used to make the classifications—associated with membership in any observed profile groups. Identifying the factors that are associated with certain wellbeing profiles in old age may be informative about what constitutes wellbeing in old age.

## Method

### Sample

Our sample consisted of members of the Lothian Birth Cohort 1936 (LBC1936, n = 1091, 548 males, 543 females), most of whom had taken part in the Scottish Mental Survey 1947 [22,23]. They formed a narrow-age cohort of individuals born in 1936 that has been extensively assessed [22,23] including screening for dementia. Recruitment took place between 2004 and 2007, when the participants had a mean age of about 70 years (mean age = 69.53, SD = 0.85). They were relatively healthy, and almost all lived independently in the community in the Edinburgh and the surrounding Lothian areas of Scotland. The study was in compliance with the Helsinki Declaration. Ethical permission was obtained from the Multi-Centre Research Ethics Committee for Scotland (MREC/01/0/56) and from the Lothian Research Ethics Committee (LREC/2003/2/29). All participants gave informed consent in writing. Full description of the recruitment and testing, including description of the tests and questionnaires used in the assessment can be found in [22].

### Procedure

Prior to describing the actual measures used, we describe how the study’s design and domains of function were conceived. In this study we addressed two important methodological issues that arise in studying differential wellbeing profiles in old age. First, mixed-age samples are usually used in this area of study [24,25]. Age and the factors contributing to stable levels of physical, emotional, and psychosocial function are confounded in such samples because, despite declining individual levels of function in all domains with age, relative levels also show considerable stability over time for both cultural and individual reasons. We minimised the interpretive difficulties this creates by studying a cohort of individuals who were all born in the same year. The second issue we addressed is to what degree profiles of wellbeing can be characterised by variables other than those variables that were already used to define the domains of wellbeing on which the profiles were based. For example, are some discrete sub-populations more representative of some socio-economic groups than others? That is, we identified *a priori* some key variables to explore whether there were separable groups with respect to physical, psychosocial and emotional wellbeing in old age in our sample; then we investigated whether the groups we identified showed important associations with another, entirely new set of variables—which we called ‘external’ because they were not used in defining the groups. We thus hypothesised that latent classes—that is, sub-populations—exist that explain the distribution of profiles and subsequently sought to characterize these latent classes using new variables. The advantage of this approach is that it allows both enumeration and detailed description of the different ways that older adults exhibit wellbeing across the physical, emotional and psychosocial domains.

From the available variables, we chose three markers to represent physical functioning: level of physical function, days per month active, and activities of daily living. These variables correlate with depression - individuals with more feelings of depression report more physical dysfunction and are less physically active [26]. Thus, we also chose expression of depression and anxiety (reversed) symptoms to represent emotional wellbeing. We chose four quality of life (QOL) domains to represent psychosocial wellbeing: physical, psychological, social, and environmental wellbeing. Because QOL is a multidimensional facet, definitions of QOL within the literature vary. Some authors [27] combine facets of emotional wellbeing, such as traits of anxiety and depression and facets of health and physical function with facets of social and environmental wellbeing to define QOL. In this study, in the measures we call ‘physical function’ we used variables relating only to someone’s self-reported activity levels and independence in ADLs (number of days active per month, intensity of physical activity and ADLs). In the QOL physical measures, participants were also asked questions relating to physical health, pain and discomfort, energy and fatigue, sleep and rest, working capacity, mobility and dependence on medication. Hence we wanted a variable relating only to activity levels and independence, setting it aside from other physically-related variables. In the measures we call ‘emotional wellbeing’ we used the Hospital Anxiety Depression Scales, which are able to indicate symptoms of anxiety and depression, thus only relating to someone’s emotive feelings; whereas, in the QOL psychological measures, participants were also asked about their thinking, learning, concentration, self-esteem, and body image. Thus the QOL variable was more thorough in assessing the participants’ overall wellbeing, whereas the other measures were more specific to emotional and physical wellbeing.

In this study we also wanted to differentiate groups based on self-rated health attitudes and behaviours, and use more objective physical scores as the external variables. We wanted the perceptions of participants’ own views on their health – self-rated QOL, emotional wellbeing, and physical activity; we used objective measures, such as grip strength and lung function, as external variables to describe the groups. The advantage of using self-rated health is that it is a predictor of mortality, even in otherwise (objectively) healthy individuals [28,29]. Therefore, all three variables – physical function, emotional wellbeing, and psychosocial wellbeing, were self-rated, thus we used participants’ own perceptions of their own physical and emotional and psychosocial wellbeing.

### Measures of wellbeing

#### Physical functioning

We used level of physical activity, total number of days active per month, and activities of daily living to derive this component. For level of physical activity participants were assessed on a 6-point scale varying from house-chores to intense exercise and for total number of days active per month participants were asked how many days they exercised vigorously. Higher figures indicated higher levels of activity in both instances. For activities of daily living, the Townsend’s scale [30] is a 9-item scale that assesses ability to perform activities of daily living involved in personal hygiene, getting dressed, eating independently, and being mobile, with answers ranging from ‘yes, with no difficulty’, to ‘yes, with some difficulty’, and ‘no, needs help’, with scores of 0, 1, and 2 respectively.

#### Emotional wellbeing

Participants completed the Hospital Anxiety and Depression Scales (HADS) [31]. This assesses recently prevailing emotional states. There are seven items for anxiety and seven items for depression, with scores ranging from 0 to 3 per item, and 0 to 21 per subscale. Higher scores signify greater anxiety and depression.

#### Quality of life

Participants completed the brief version of the World Health Organisation Quality of Life Assessment [32]. This measures quality of life in four subscales covering physical, psychological, social, and environmental domains, and eight sections covering, mental health, social, emotional, and physical role functioning, general health perceptions, bodily pain, physical function, and vitality. There are 26 questions in all. All items are measured on a five-point scale, with higher scores denoting better quality of life. This questionnaire has good validity, reliability and consistency, and is applicable cross-culturally [32].

### External covariates

#### Demographic measures

These included: self-reported total number of years in formal education; marital status (i.e., single, married, widowed, separated, or divorced); living status (i.e., alone or not alone); and the person’s own highest professional social class during working life. For females, husband’s social class was used when higher.

#### Prior cognitive ability

The *Moray House Test No. 12*[33,34] administered when participants were aged about 11 years, on 4^th^ June 1947 in the Scottish Mental Survey 1947, is a group-administered test of general cognitive ability. *The National Adult Reading Test (NART)*[35] is a widely-used test to estimate prior cognitive ability. It requires the participant to read 50 irregular English words.

#### Current cognitive ability

We used three cognitive scores derived from principal components analyses (PCA) to represent general cognitive ability (*g*), memory, and speed. For *g* we used six Wechsler Adult Intelligent Scale-III^UK^ (WAIS-III) [36] subtest scores; these included *Symbol Search* (speed of information processing); *Digit-Symbol Coding* (speed of information processing); *Matrix Reasoning* (non-verbal reasoning); *Digit-Span Backwards* (working memory*); Letter-Number Sequencing* (working memory); and *Block-Design* (constructional ability). For the derived memory component we used four subtests from the Wechsler Memory Scale-III^UK^ (WMS-III) [37], which included *Logical Memory I* (immediate recall of verbal declarative memory); *Logical Memory II* (delayed recall of verbal declarative memory); *Verbal Paired Associates I* (immediate verbal learning memory); and *Verbal Paired Associates II* (delayed verbal learning memory). Speed of processing tests to derive the speed component included means and standard deviations of Simple Reaction Time (SRT); Choice Reaction Tim*e* (CRT); [38,39]; and *Inspection Time* (IT) (non-speeded elementary visual processing assessed on a computer) [40].

#### Personality measures

Participants completed the *NEO Five Factor Inventory*[41] which is a self-rated 60-item Likert scale ranging from strongly agree to strongly disagree, assessing the five major personality factors: Neuroticism, Extraversion, Openness to experience, Agreeableness, and Conscientiousness.

#### General health measures

Participants were given a physical examination, which included: time to walk 6 meters; mean grip-strength of both left and right hands; lung function assessed as the best of three in forced expiratory volume in 1 second (FEV_1_) and forced vital capacity (FVC); body mass index (BMI); and systolic and diastolic blood pressure. They were tested for the *APOE* e4 allele. Participants were also asked about total units of alcohol consumed per week; and whether they currently smoked, had quit smoking, or never smoked (smoking status). It should be noted that we differentiated between the physical fitness measures here and the physical function ones used as part of the wellbeing domains to create groups. The physical fitness variables (grip strength, 6-meter walk-time, FEV_1,_ and FVC) making up the external variables used here, are bodily assessments and objective measures of wellbeing, by contrast with physical health behaviour and autonomy, such as level and intensity of physical exercise and ADLs, which are self-reported and more under the individual’s control on a daily basis.

#### Disease measures

As part of a structured interview, participants were asked for their history of cardiovascular disease (CVD) and stroke, and if they had any blood circulation problems, for their total numbers of diagnosed medical conditions, and the current total numbers of prescription medications they took.

### Statistical analyses

#### Principal components analysis

To reduce the number of variables under consideration, we extracted principal components for Physical Function and Quality of Life. We used the Statistical Package for the Social Science (SPSS, version 17.0, SPSS Inc., Chicago, IL, USA) to carry out the PCA.

One component explained 52% of the variance in the three physical function measures, on which each loaded .53 or more, which meant that each variable in the PCA contributed at least .53 to the component score. We used this component score to represent physical function in our subsequent analyses. For the four QOL subtests, one component explained 60% of the variance, on which each variable also loaded over .53 on this component, and we used the component score to represent quality of life in our analyses.

For Emotional Wellbeing, the two subscales of anxiety and depression from the HADS correlated .37 (*p* < .001). Because PCA generally requires at least 3 variables, we standardized the two sub-scores and calculated their mean. These scores were reversed so that higher scores represented more positive Emotional Wellbeing.

Data for participants whose component scores were more than 3 standard deviations form the mean were trimmed to 3 or -3 as relevant and retained in the database.

#### Latent class analysis (LCA)

We explored possible subgroups within the data, using LCA implemented in Mplus [42] LCA is a latent general mixture modelling (GMM) technique that produces the number of classes specified by the user. Although it is primarily intended for qualitative and categorical outcomes [43], it has also been used with continuous variables [44]. In this study we used LCA as a descriptive tool, and we were not expecting to find categorically distinct classes; therefore, we refer to the results of our LCA as ‘groups’ rather than ‘classes’, because it was our judgment that the analyses would not reveal naturally-occurring, entirely distinct categories of individuals but, instead, more loosely-structured sets with fuzzy boundaries that would likely shift from sample to sample. Thus, in these circumstances, the method had primarily practical and descriptive value, especially when its results could be associated with other variables involved in important life outcomes. We dealt with missing data by using the maximum likelihood estimation missing data feature in MPlus to include all participants.

We used our physical function, emotional wellbeing, and quality of life variables to estimate 2, 3, 4, 5, and 6-group LCA solutions, and evaluated their relative appropriateness in order to select the solution that appeared to specify the most appropriate number of groups. The first consideration in doing this is generally to examine model-fit statistics, which describe how well a model fits the data by summarising the discrepancy between observed and expected values. There are many such statistics available, and they do not always specify a single best-fitting model [45,46]. Two in common usage, and which we also use here, are the Bayesian Information Criterion (BIC) [47] and entropy (ENT) [48]. Typically a smaller BIC indicates a better fit; however, usually, when data are continuous, the larger the number of groups, the better the fit; hence there is a trade-off between parsimony and fit [47,49,50]. ENT indicates how well the variables predict group membership [48]. ENTs close to 1 indicate that most participants have single classes with high probabilities of membership. We avoided solutions that included groups containing less than 5% of participants, unless such a group had distinctive qualities setting it aside from the other groups. We also sought the most parsimonious solution that met our other criteria.

#### Associations with external variables

We applied analyses of variance (ANOVA) with group membership as the independent variable to describe how the groups differed from each other on variables other than those that were used to form the classes. These external variables—demographic, personality, cognitive, physical function, and disease, as described above—were used as dependent variables to test associations and thus more richly characterise the groups identified. We applied post-hoc tests for significant findings using Tukey’s Honestly Significant Difference (HSD) test comparisons in order to find out which groups differed significantly from the others. We used the SDs of the largest group (the High Wellbeing group) as the base for calculating the effect size, using Cohen’s *d*, which is the standardised difference between two means (calculated by dividing the mean differences by the pooled standard deviation to give measures of the strength of the mean differences between two variables) for all external variables between the High Wellbeing group’s scores and scores from each of the other 4 groups. We did not adjust significance levels for multiple testing in any of these analyses.

## Results

### Profile membership

Raw means and standard deviations in the whole sample for the variables that were used in the LCA can be seen in Table 1. Table 2 shows the BIC values for the latent class models, indicating minimisation of the BIC at five groups. The ENT had a maximum of .825 at 2 groups and a minimum of .653 at 4 groups. The 3- and 6-group solutions had ENTs of .715 and .706, whereas 5 groups had an ENT of .694. The 2-group solution showed the best discrimination amongst groups, whereas the rest seemed to average at an ENT of .7. The 6-group solution contained groups with less than 5% of the population, and the 4-group solution had the lowest ENT and a higher BIC than the 5-group solution. The 5-group solution was deemed the most suitable because its BIC was optimal and the groups appeared to have tractable characteristics. Most likely group membership probabilities ranged from .71 to .86, indicating reasonably clear group membership for most participants. We used this solution for further description and comparisons. 

**Table 1:** Physical, Emotional and Psychosocial wellbeing variable means

**Variable**	**Ranges**	**Total participants, n = 1091**
**Physical Function**		
Level of physical activity	1.1-1.5	2.98 (1.1)
Days active per month	0-31	7.68 (8.1)
ADLs	0-2	.99 (2.0)
**Emotional Wellbeing**		
HADS (Anxiety)	0-21	4.89 (3.2)
HADS (Depression)	0-21	2.80 (2.2)
**Quality of life**		
Physical QOL	0-20	16.10 (2.6)
Psychological QOL	0-20	15.67 (1.8)
Social QOL	0-20	17.14 (2.4)
Environmental QOL	0.20	16.71 (1.8)

Standard deviations in parentheses.

*Note.* ADLs = Activities of Daily Living. HADS = Hospital Anxiety and Depression scales. QOL = Quality of Life. Higher scores denote better wellbeing, ADLs and HADS scores were reversed to equate a higher score with better wellbeing.

**Table 2:** Model information criteria for each of the four, five and six group solutions

**Group-solution**	**BIC**
Two	8155.96
Three	8073.56
Four	8038.90
Five	8026.98
Six	8045.79

Note. BIC = Bayesian information criterion.

**Figure 1:**
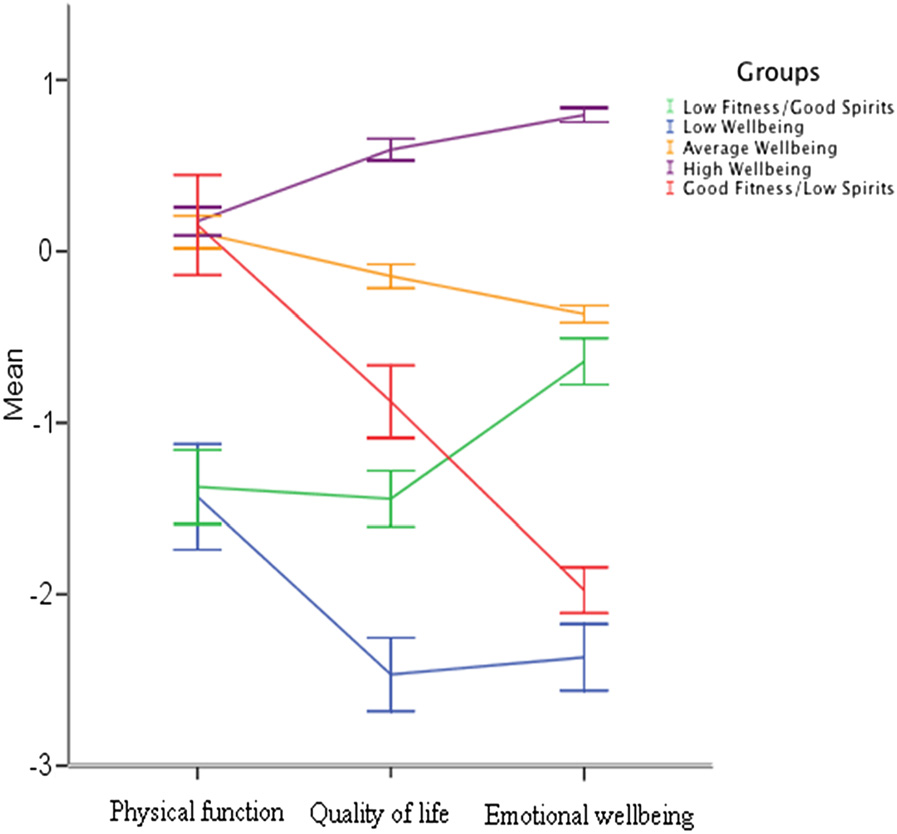
The groups’ mean scores on each of the psychosocial factors, namely physical function, quality of life, and emotional wellbeing, with standard error bars at 2 standard errors, as generated from latent class analysis for the LBC 1936 sample.

We labelled the group comprising the majority of the sample (n = 515, 47.2%) High Wellbeing as they tended to score relatively highly across all three domains. We also labelled groups representing Average Wellbeing (n = 417, 38.3%) and Poor Wellbeing (n = 37, 3.4%), reflecting generally those overall levels of function. There were contrasting patterns of wellbeing across domains in the two final groups: one group was physically fit but had relatively low Emotional Wellbeing (n = 60, 5.5%), which we labelled Good Fitness/Low Spirits. Another was in relatively poor physical condition but showed relatively good emotional wellbeing (n = 62, 5.7%), which we labelled Low Fitness/Good Spirits. The groups’ means on each of the components of Physical Function, Quality of Life, and Emotional Wellbeing are illustrated in Figure 1; there, one can see three groups that scored relatively high, average, or low across all domains, and another two groups that displayed an interaction between Emotional Wellbeing and Quality of Life.

### Profile characteristics

**Figure 2:**
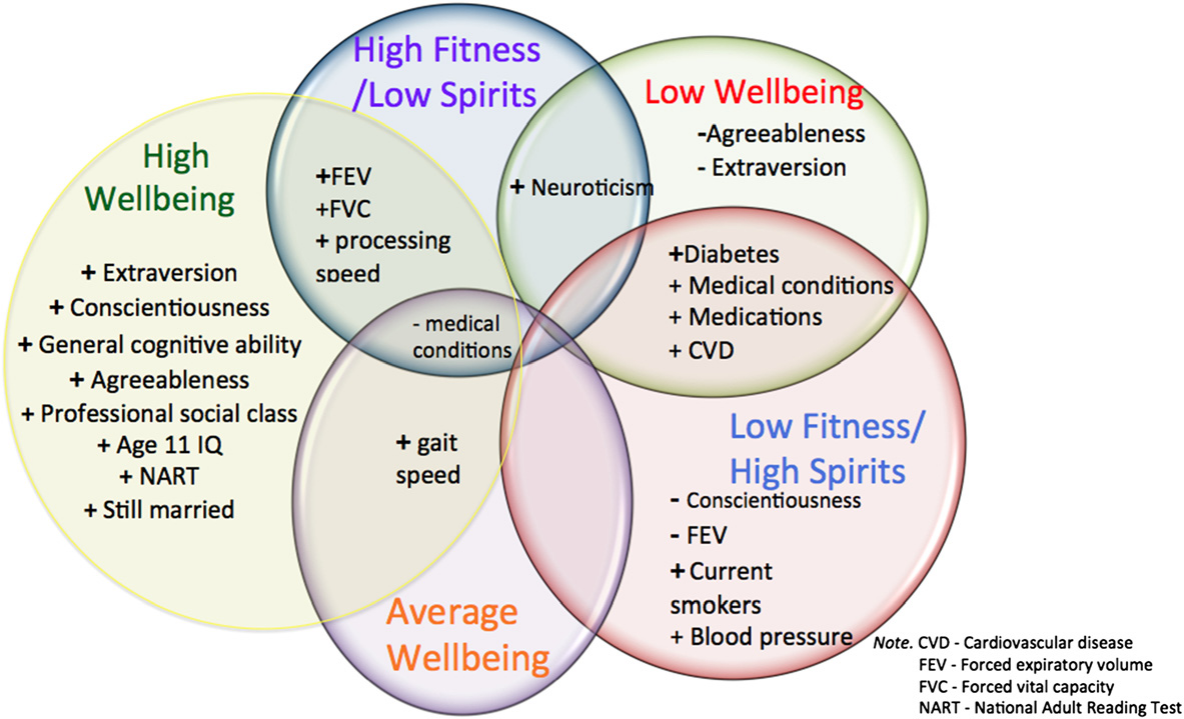
Venn diagram displaying common and distinctive significant features characterising the groups.

Because this section of the Results contains so many comparisons, we provide, at the start, a Venn diagram illustrating the main similarities and differences amongst the groups on the external variables (Figure 2). The + signs in the diagram indicate significant high scores and the – signs indicate significant low scores on the variables in that particular group in comparison to the mean of each of the other groups, or to at least one particular group’s mean as specified in more detail in the results below. The diagram shows, as a summary, that personality and disease indices played a significant role in distinguishing amongst the groups. Table 3 shows the means and standard deviations for each group on external variable scores. 

**Table 3:** External variable means and significance values for each of the five groups

	**Latent groups**					
**Variable**	**High Wellbeing (n = 515)**	**Average Wellbeing (n = 417)**	**Low Wellbeing (n = 37)**	**Good Fitness/Low Spirits (n = 60)**	**Low Fitness/Good Spirits (n = 62)**	***P***
**Demographics**						
Number of males (%)	280 (54.5)	196 (47.1)	16 (48.5)	27 (45.0)	28 (45.2)	.880
Age 11 IQ	101.9 (13.9)	99.5 (14.5)	97.7 (17.4)	93.0 (18.6)	95.2 (17.6)	.001
NART (range 0-50)	35.3 (7.8)	34.1 (8.3)	34.1 (7.2)	32.1 (9.3)	32.6 (9.0)	.007
Yrs. Educ. (range 7-14 years)	10.8 (1.1)	10.7 (1.2)	10.4 (.09)	10.5 (1.2)	10.6 (1.0)	.030
Number married (%)	375 (73)	298 (71.6)	19 (51.4)	42 (70)	39 (62.9)	.001
Number living alone (%)	126 (24.5)	88 (21.2)	12 (32.4)	18 (30)	22 (35.5)	.001
Number in the professional social class (%)	104 (20.6)	63 (15.3)	6 (17.1)	9 (15.8)	8 (13.3)	.001
**Personality (range 0-60)**						
Neuroticism	24.97 (5.7)	31.21 (6.4)	40.15 (6.4)	38.4 (6.7)	34.4 (7.3)	.001
Extraversion	40.88 (5.4)	38.04 (5.7)	32.13 (6.7)	36.1 (4.8)	35.90 (5.1)	.001
Openness	38.53 (5.58)	37.70 (6.06)	37.27 (5.63)	37.50 (5.52)	37.37 (6.17)	.183
Agreeableness	46.25 (5.2)	45.47 (5.2)	42.64 (4.3)	44.08 (5.0)	43.13 (5.0)	.001
Conscientiousness	48.19 (5.6)	46.27 (5.8)	44.72 (7.05)	42.94 (5.6)	41.37 (6.8)	.001
**Physical function and health (range for each variable in parentheses)**						
Grip strength (4-60kg)	.05 (1.17)	-.06 (.26)	.31(3.05)	-.09 (.27)	-.11(.29)	.101
6m walk time (1.05 – 14.74m)	.16 (.7)	.05 (.90)	−1.19 (2.0)	.19 (1.4)	.85 (1.4)	.001
FEV_1_ (.49 – 5.13)	.11 (1.0)	-.00 (.9)	-.40 (1.0)	-.18 (1.2)	-.47 (1.0)	.001
FVC (1.13– 6.93)	.10 (1.0)	.03 (1.0)	-.33 (.9)	-.35 (1.0)	-.52 (.9)	.001
BMI (16.02 - 48.52)	27.61 (4.1)	27.75 (4.5)	29.35 (5.3)	27.76 (4.8)	28.98 (5.2)	.044
Units of alcohol/week (0-140)	10.80 (12.9)	11.07 (15.7)	9.19 (17.5)	9.08 (9.4)	9.19 (17.5)	.080
Number of current smokers (%)	61 (11.9)	52 (12.5)	9 (24.3)	7 (11.7)	17 (27.4)	.001
*APOE* e4 present (%)	158 (32.8)	106 (22.9)	10 (33)	15 (27.8)	17 (27.4)	.421
**Disease (range in parentheses)**						
Number with high blood pressure (%)	187 (36.4)	172 (41.3)	20 (54.1)	17 (28.3)	35 (56.5)	.002
Number with diabetes (%)	27 (5.3)	34 (8.2)	13 (35.1)	4 (6.7)	13 (21)	.001
Number with CVD (%)	108 (21)	99 (23.8)	19 (51.4)	18 (70)	23 (37.1)	.001
Number with blood circulation problems (%)	68 (13.2)	58 (14)	7 (18.9)	7 (11.7)	7 (18.9)	.082
Number with history of stroke (%)	16 (3.1)	22 (5.3)	4 (10.8)	6 (10)	6 (9.7)	.024
Total number of medications (0-8)	2.52 (2.30)	2.99 (2.41)	5.43 (2.62)	3.18 (2.81)	4.82 (2.80)	.001
Total medical conditions (0-8)	2.76 (1.6)	3.37 (1.7)	5.17 (1.9)	3.07 (1.7)	4.42 (1.7)	.001
**Cognition (range in SD units in parentheses)**						
*g* (2.51-2.11)	.13 (.7)	-.04 (.7)	-.45 (.8)	-.23 (.7)	-.24 (.7)	.001
Memory (-2.78-1.78)	.07 (.79)	-.02 (.82)	-.05 (.84)	-.21 (.92)	-.04(.82)	.093
Speed (-3.00 – 1.86)	-.04 (.5)	-.01 (.6)	-.26 (.7)	.17 (.6)	-.14 (.4)	.002

Standard deviations in parentheses.

Note. IQ =Intelligence Quotient. NART = National Adult Reading Test. Yrs Edu = Total number of years in formal education.

FEV_1 =_*Forced expiatory volume in 1 second. FVC = Forced vital capacity. BMI = Body Mass Index. APOE e4 = Apolipoprotein E allele e4. CVD = cardiovascular disease. No adjustments for multiple testing were made.*

### The High Wellbeing group

The majority of individuals (73.0%) in the High Wellbeing group were still married, as opposed to being widowed, separated or divorced; significant differences (p < .05) were present between the High Wellbeing group and the Low Wellbeing group (only 51.4% were still married in this group). A significantly higher number of individuals (20.6%) in the High Wellbeing group belonged to the professional social class, as opposed to individuals in the Low Fitness/Good Spirits group (6.0%). This group also had a significantly higher age-11 IQ, higher NART scores, a higher *g*, and faster processing speed than the rest of the groups.

### The Average Wellbeing group

The Average Wellbeing group was on average, doing relatively well across most measures. Although this group was not distinguished markedly from the rest since it scored averagely, this group, along with the High Wellbeing group, had more positive characteristics than the rest of the groups, namely fast gait speed, average prior and current cognitive ability, and low disease rate. With regards to personality it showed high mean scores on traits of Extraversion, Conscientiousness, Agreeableness, and low mean scores on Neuroticism.

### The Low Wellbeing group

\The Low Wellbeing group had a higher percentage of divorce than the rest of the groups, showing a significant difference from the High Wellbeing group (13.5% vs. 6.8%). The Low Wellbeing group scored significantly highly on Neuroticism (*d* = 2.38), and low on Agreeableness (*d* = 0.93) and Extraversion (*d* = 1.45) compared to the rest of the groups. The highest percentage of individuals with diabetes was found in this group (35.1%), which significantly differed (*p* < .001) from the Average Wellbeing group (8.2%), the High Wellbeing group (5.3%), and the Good Fitness/Low spirits group (6.7%). Individuals in this group also had the highest percentage (51.4%) of history of CVD, showing significant differences from the Average Wellbeing (23.8%, p < .01) and the High Wellbeing group (21%, *p* < .001). The Low Wellbeing group also had the highest mean scores on total number of medications taken (5.43) and number of diagnosed medical conditions (5.17), which significantly differed from the rest of the groups with effect sizes of 1.20 and 1.53 respectively.

### The Low Fitness/Good Spirits group

This group had significantly lower mean scores on Conscientiousness scores than the rest of the groups (d = 1.14). The Low Fitness/Good Spirits group had a higher percentage of smokers (27.4%) than the rest of the groups, which differed significantly (*p* < .01) from the Average Wellbeing (12.5%) and the High Wellbeing (11.9%) groups. The Low Fitness/Good Spirits group had the highest percentage (56.5%) of individuals with high blood pressure. This differed significantly (*p* < .05) from the High Wellbeing group (36.4%) and the Good Fitness/Low Spirits group (28.3%). Along with the Low Wellbeing group, the Low Fitness/Good Spirits group also had a high percentage of individuals with diabetes (21%), showing significant differences from the Average Wellbeing group (*p* < .01), the High Wellbeing group (at *p* < .001, *d* = .48), and the Good Fitness/Low Spirits group (at p < .05). Significant differences (*p* < .05) were also present between the High Wellbeing group and the Low Fitness/Good Spirits group. The Low Fitness/Good Spirits group had the lowest mean scores on Contentiousness, and also contained the highest percentage of current smokers and had low mean scores on physical function. This group also had the lowest levels of FEV_1_ and FVC, with effect sizes ranging from 0.53 to 0.67 with respect to the other groups. This was not surprising given their smoking and physical function status. It seemed that this group’s low mean score on Conscientiousness was reflected in its behaviour - it was one of the least healthy, but still had relatively high spirits in comparison to the Good Fitness/Low Spirits and the Low Wellbeing group.

### The Good Fitness/Low Spirits group

The Good Fitness/Low Spirits group was marked by its significantly high mean score on the Neuroticism trait, which was only second to the Low Wellbeing group. It also had a higher mean FEV_1_ and a faster mean processing speed than the Low Wellbeing and the Low Fitness/Good Spirits groups.

## Discussion

We used data from the Lothian Birth Cohort 1936 to explore group profiles of physical, psychosocial and emotional domains of function among 70-year-old individuals. Our results indicated that, although wellbeing across these domains was, by and large, unidimensional (i.e. ranging from low to high wellbeing as illustrated by the High, Average, and Poor Wellbeing groups), some individuals in our sample seemed to be in relatively good physical condition but still to experience emotional stress, and some individuals appeared to be in relatively poor physical condition, yet were relatively satisfied with their situations (as illustrated by the Good Fitness/Poor Spirits and the Poor Fitness/Good Spirits groups).

Consistent with the literature on young old age and the overall health screening that went into sample recruitment, [17,24] our largest group (the High Wellbeing group) scored relatively highly in the physical, psychosocial, and emotional domains of wellbeing we considered, indicating that the majority of participants were doing reasonably well. However, the disparities present in the Good Fitness/Poor Spirits and the Poor Fitness/Good Spirits groups were consistent with some previous studies that also have focused on such differences [17,24,25,51] emphasising that the associations among physical function, emotional stability and quality of life typically depicted in the literature [52,53] are never complete.

We then used the generated group profiles to assess differences arising amongst groups using a wide range of variables related to later-life wellbeing. The results indicated personality traits, specifically Neuroticism (effect sizes ranging from 0.45 to 2.16) and Conscientiousness (effect sizes ranging from 0.22 to 1.14), as the strongest discriminators among the profiles.

The groups that had higher Neuroticism scores, specifically the Low Wellbeing and the Good Fitness/Low Spirits groups, also had more diagnosed medical conditions and were taking more medications. Although the literature indicates that, overall, individuals with high physical functioning generally tend to score low in Neuroticism and have relatively high spirits [54] this overall observation may conceal underlying subgroups with different characteristics, as our results from the Good Fitness/Low Spirits group indicated. This group in fact had significantly high levels of Neuroticism and significantly low levels of Extraversion a result that reflected the well-known association between Neuroticism and depression [54]. This may have reflected environmental surroundings, social ties, and levels of perceived support. For example, there is evidence that individuals with physical disability but with supportive environments who are resilient, tolerant of negative change, and have positive attitudes are less likely to feel depressed than physically fit individuals who do not have these characteristics [2,55].

### Strengths and limitations

A strength of the study was that all participants were born in the same year (1936); thus all participants had the same chronological age, which eliminated age cohort effects. Furthermore, to characterise any distinct wellbeing patterns more fully, the LBC1936 have provided data ranging from childhood IQ (the validated and reliable MHT given when the participants were aged 11), to current cognitive tests (the WAIS -III and WMS-III), and health and lifestyle indices, making it possible to examine a broad range of external associations and to examine factors involved in lifetime cognitive change. Such a range of variables can be helpful in identifying differences relating to specific groups of individuals. Studies with limited numbers of variables may be unable to provide comprehensive explanations of differences among groups.

The cohort used in this study was relatively healthy. This is common when studying 70-year old individuals who volunteer for research, and who have been screened for dementia. It is possible that we missed some parts of the wellbeing continuum, or even separable groups of individuals, due to the relatively high health status present in this cohort. In fact, although we tried avoiding groups containing less that 5% of the whole sample, some groups in the study consisted of small numbers of individuals that seemed to contain distinctive qualities setting them aside from the rest of the groups; e.g., the Low Wellbeing group, which only contained 3.4% (n = 37) of the sample. This is also sometimes an indication that the LCA analytic procedure has capitalised on chance gaps in otherwise continuous data, especially when the small groups tend to fall at the extremes of the distributions of the defining variables, as was the case here.

We used the HADS to assess the emotional wellbeing of the cohort; however, we acknowledge that absence of depression and/or anxiety symptomatology alone does not necessarily mean high emotional wellbeing, and there is more to emotional integrity. We used these scales as a measure reflecting only lack of depression/anxiety.

Finally, in our study we applied LCA to continuous data as have a number of others [50,56] thus we did not expect to find naturally occurring distinct classes of individuals, but used the technique to explore and describe profiles of physical and psychosocial wellbeing and to measure associations between those profiles and life outcome variables. Previous studies [17,25,57] that have looked at profiles of functioning across individuals have typically focused on cluster analysis to characterise group patterns, which is a more subjective way of classifying individuals, and is subject to similar limitations when applied to continuous data, as has often been the case. Although LCA does not allow hierarchical grouping, it has advantages over other methods due to its objective measures of model fit criteria, maximum likelihood estimation and group-membership probabilities. We applied no corrections in our tests of group differences for the multiple tests we carried out. This may have given rise to Type I errors.

Although this study was cross-sectional, the LBC1936 is an ongoing study, with future opportunities to follow up the current results on groups’ stabilities and developmental patterns longitudinally.

## Conclusion

Results from this study indicated that wellbeing in old age is not necessarily an all-or-nothing phenomenon; rather, individuals can show relatively high wellbeing patterns in one area despite relatively poor functioning in other areas. Our study supports previous research findings [24,25] demonstrating uneven profiles of function within individuals. Ultimately, the results from this study highlight the importance of this type of research when considering recent revisions to the definition of what makes aging successful by highlighting the possibility that different people can age successfully in different ways.

Longitudinal data are necessary for future research to follow up on developmental patterns that define successful physical, emotional and psychosocial aging. Studies from other cohorts and in this cohort over time will be important in revealing how results may vary. Many older people rate themselves as aging successfully, even if they do not meet objective criteria in areas such as physical function [2]. Such a subjective approach would possibly give a different perspective on how individuals may be grouped on wellbeing in old age.

## Competing interests

The authors declare that they have no competing interests.

## Authors’ contributions

ARZ, IJD, JMS and WJ have taken part in the designing and planning of the study. ARZ drafted the manuscript, while JMS, WJ and IJD contributed to pre-submission drafts, revisions and edits of the manuscript. All authors have read and approve the publication of the final manuscript.

## Pre-publication history

The pre-publication history for this paper can be accessed here:

http://www.biomedcentral.com/1471-2318/12/64/prepub
